# Exploring Viewing Behavior Data from Whole Slide Images to Predict Correctness of Students' Answers during Practical Exams in Oral Pathology

**DOI:** 10.1155/2014/706470

**Published:** 2014-11-26

**Authors:** Slawomir Walkowski, Mikael Lundin, Janusz Szymas, Johan Lundin

**Affiliations:** ^1^Poznan University of Technology, M. Sklodowska-Curie Square 5, 60-965 Poznan, Poland; ^2^Institute for Molecular Medicine Finland (FIMM), University of Helsinki, P.O. Box 20, 00014 Helsinki, Finland; ^3^Department of Clinical Pathology, Poznan University of Medical Sciences, Przybyszewski Street 49, 60-355 Poznan, Poland

## Background

The use of whole slide images (WSIs) allows tracking and recording how a histological slide is viewed. Gathered data about viewing behavior while interpreting WSIs may result in a variety of analyses. When the tracking is done during an exam, we can discover how students view WSIs. Moreover, we may try to correlate their way of viewing slides with correctness of the answers they give. Particularly, we can potentially find out to what extent a specific viewing behavior is likely to result in a correct or incorrect answer from a student.

## Method

To record viewing behavior, we utilized a software-based view path tracking method, which does not require any specialized equipment. It gathers information about subsequently viewed fragments (view fields) of WSIs. The method was used during exams in oral pathology in Poznan University of Medical Sciences in Poznan, Poland, in years 2012-2013. Each dental student was given 50 exam questions with a WSI attached to each of them. The students were informed about and agreed on the tracking. Stored data and further analysis results are anonymous and so far without any impact on the final students' evaluation and scores. The WSI viewing system used during the exam was WebMicroscope (Fimmic Ltd, Helsinki, Finland) and the view path tracking method is an integrated, but optional, part of it. In total, we collected information about approximately 130,000 view fields coming from about 180 dental students viewing WSIs during the exams. Gathered data was analyzed numerically, with some help from generated visualizations. A set of statistics was calculated per student per question and it included, for example, number of view fields, magnification level, and dispersion of view fields. Statistical methods were used to assess the correlation between calculated metrics and correctness of students' answers. We also utilized machine learning approaches to check to what extent viewing behavior data can be used to predict a correct or incorrect answer coming from a student. For this purpose, we used gathered and processed data as a labeled set of instances.

## Results

Two exams were successfully conducted with the view path tracking turned on, which resulted in a dataset covering students' WSI viewing behavior. The aggregated metrics depicted certain viewing patterns. Analysis of the calculated statistics allowed finding some correlations between metrics values and exam answers. When used as features for machine learning, the metrics helped estimate probabilities of answers correctness.

## Conclusion

Software-based view path tracking appears to be a useful method of discovering WSI viewing behavior and investigating decision making process of dental students who take a practical exam in oral pathology. Analysis of collected data provides interesting insights into how the slides are viewed, how the viewing patterns correlate with students' answers, and what the potential of the view path tracking data is when predicting correctness of the answers.

